# CTLA-4 Haploinsufficiency Presenting as Extensive Enteropathy in a Patient With Very Early Onset Inflammatory Bowel Disease

**DOI:** 10.1097/PG9.0000000000000099

**Published:** 2021-07-12

**Authors:** Ngoc N. Tran, Mala Setty, Elaine Cham, Alice Y. Chan, Sabina Ali

**Affiliations:** From the * Department of Pediatrics, UCSF Benioff Children’s Hospital Oakland, Oakland, CA; † Division of Pediatric Gastroenterology; ‡ Department of Pathology and Laboratory Medicine, UCSF Benioff Children’s Hospital Oakland, Oakland, CA; § Department of Pediatrics UCSF Benioff Children’s Hospital, San Francisco, CA; ‖ Division of Pediatric Allergy, Immunology, and Bone Marrow Transplant.

**Keywords:** abatacept, cytotoxic T-lymphocyte-associated protein 4 haploinsufficiency, immune dysregulation, enteropathy, immunodeficiency, autoimmunity, VEO-IBD, inflammatory bowel disease

## Abstract

Patients with very early onset inflammatory bowel disease (VEO-IBD) have a higher incidence of monogenic disease compared to older age groups. Age, alone, is a strong predictor for monogenic disease. We discuss a case of VEO-IBD in which the patient presented with severe and refractory enteropathy, leading to diagnosis of CTLA-4 haploinsufficiency. Genetic workup showed de novo heterozygous deletions of the CTLA-4 and ICOS genes. This case was unique, as the patient did not have the other manifestations commonly present with the disease. We advocate for early and routine genetic workup of VEO-IBD, as patients with monogenic IBD have high morbidity and mortality, if inadequately treated. Our patient did not respond to conventional treatment modalities and required targeted treatment with Abatacept, a CTLA-4 agonist.

## INTRODUCTION

Very early onset inflammatory bowel disease (VEO-IBD) is defined as IBD with age of onset of <6 years old. Unique features include positive family history, severe disease course, and resistance to standard treatment.^1^ Patients can have a higher incidence of autoimmunity, primary immunodeficiency, and lymphoproliferative disease.^2^ Within the VEO-IBD age group, there is an enrichment of monogenic causes, although studies have shown that the detection of known monogenic causes can be quite variable (0%–33%)^3^. Monogenic defects lead to disease via disruption of the intestinal epithelial barrier, neutrophil activity, T- and B-cell differentiation, regulatory T-cell activity, or hyper and autoinflammatory responses.^2^

CTLA-4 haploinsufficiency (CHAI) is a primary immune system disorder. CTLA-4, an inhibitory receptor found on regulatory T cells, plays a role in maintaining immune homeostasis and self-tolerance. CTLA-4 suppresses T cell activation by competing with CD28 for binding of the ligands CD80 and CD86, which are expressed on antigen-presenting cells.^4^ Similar to deficient humans, CTLA-4 mouse models exhibit impaired Treg cell suppressive function, T-cell hyperproliferation, severe autoimmune disease, and early mortality.^5^ We describe the diagnosis of CHAI in a 6-year-old female who presented with severe refractory enteropathy and was found to have de novo heterozygous deletions of the CTLA-4 and ICOS genes.

## CASE REPORT

**FIGURE 1.**
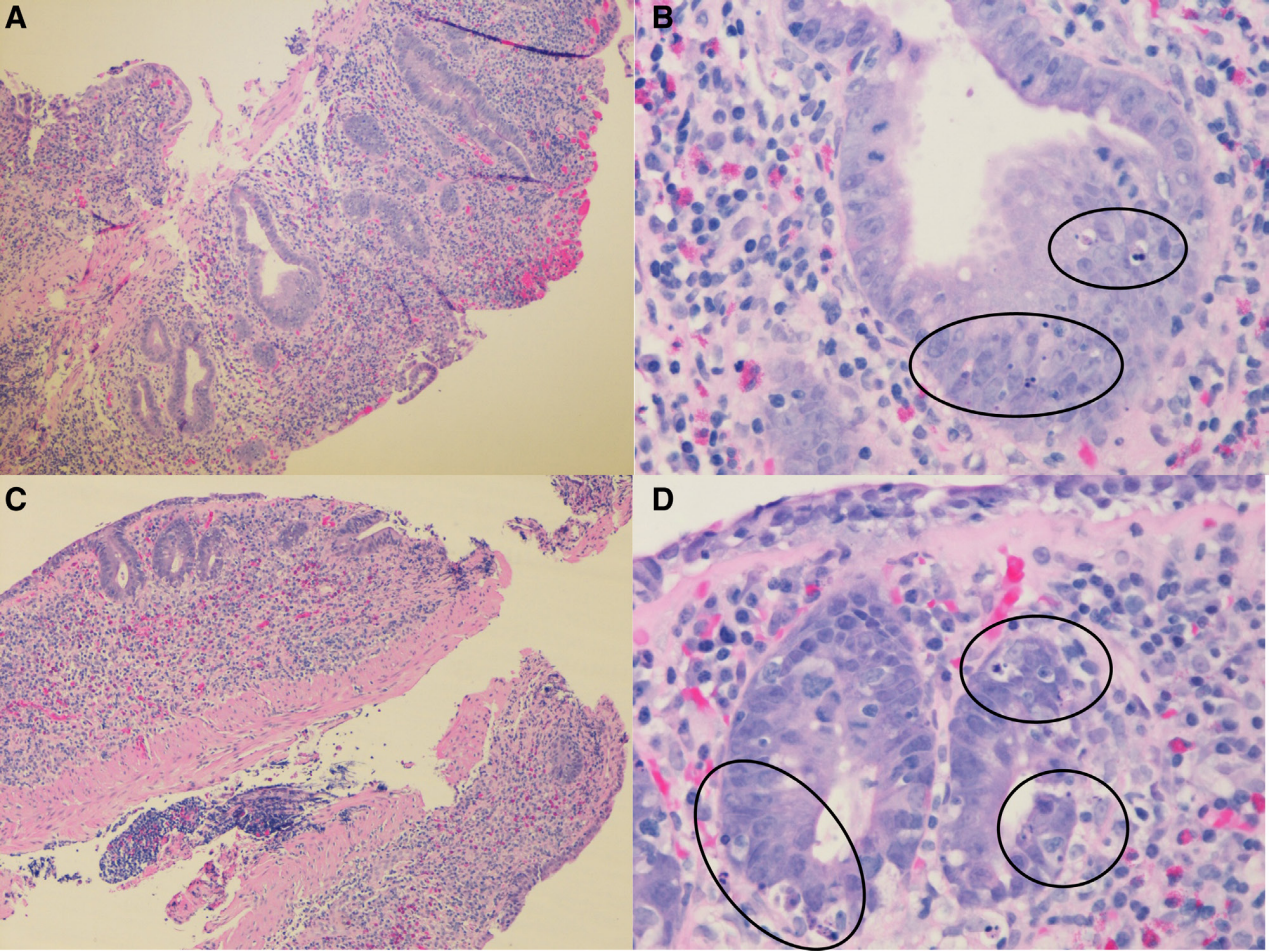
Duodenum and colon biopsies. A) Duodenum biopsy shows flattened villi and distorted crypts (H&E, original image 10× magnification). B) High power field of duodenum biopsy shows numerous apoptotic bodies in the crypts (H&E, original image 50× magnification). C) Colon biopsy shows marked crypt loss (H&E, original image 10× magnification). D) High power field of colon biopsy shows many apoptotic bodies (H&E, original image 50× magnification).

**FIGURE 2.**
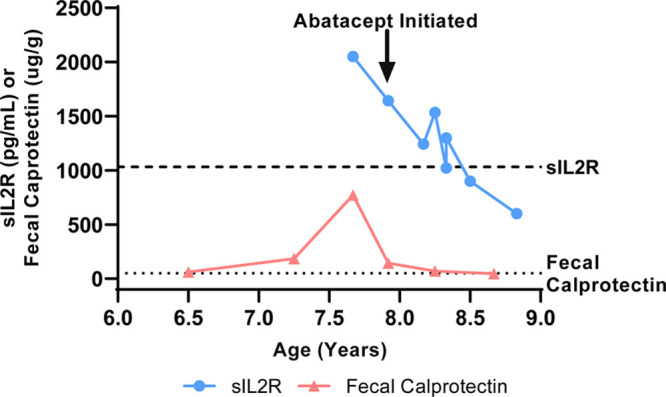
The graph shows resolution of disease markers after the initiation of Abatacept. The graph plots both sIL2R (circles) and fecal calprotectin (triangles) levels over the disease course. The arrow illustrates the initiation of abatacept. The dashed and dotted lines represent the upper limit of normal for sIL2R (<1033 pg/mL) and fecal calprotectin (<50 µg/g), respectively.

A 6-year-old Hispanic female with speech delay was admitted for malnutrition, diarrhea, and abdominal pain. She had 3 years of diarrhea, in addition to increased stool urgency, stool incontinence, hematochezia, abdominal pain, distension, and bloating. There was no family history of consanguinity, IBD, autoimmunity, or immunodeficiency. Laboratory results showed Hgb 10.9 gm/dL, MCV 71 fL, CRP 14.4 mg/L (normal < 5mg/L), ESR 29 mm/hr (normal < 13 mm/hr), and IgG 1798 mg/dL (normal 593–1723 mg/dL). The IgA level was undetectable; therefore, her normal celiac panel was uninterpretable. Fecal calprotectin was 61 µg/g (normal < 50 µg/g). Antienterocyte antibody, thyroid studies, hepatitis panel, and stool infectious studies were negative. Her upper and lower endoscopy showed fissured, thickened, and nodular mucosa throughout the stomach, duodenum, colon, and rectum, with a polyp in the sigmoid colon. Colonic biopsy showed mild colitis with apoptotic bodies and duodenal biopsy showed severe villous blunting with acute cryptitis. MR enterography was normal. Due to the pathology findings and IgA deficiency, there was a concern for celiac disease versus IBD. She was discharged on sulfasalazine, a gluten-free diet, nasogastric tube feeds, and periactin. Despite medical intervention, she had ongoing symptoms and was readmitted for intravenous steroid treatment. She was admitted a third time for gastric tube placement and repeat endoscopy. Calprotectin increased to 773 µg/g and soluble IL2R (sIL2R) was 2051 pg/mL (normal < 1033 pg/mL). She was treated for a urinary tract infection. Other infection studies including gastric viral panel, bacterial stool culture, stool ova and parasite, and giardia antigen were negative. Pathology showed gastritis, marked chronic duodenitis with an increase in apoptotic bodies, and marked chronic colitis with acute inflammation, increase in apoptotic bodies, and crypt remodeling and loss (Fig. 1). She was started on total parenteral nutrition (TPN) to supplement her nutrition. Due to her age and severe findings, an immunology evaluation was initiated. Lymphocyte subsets, including B cell count, tetanus and pneumococcal titers, and neutrophil oxidative index test were nonrevealing. A targeted primary immunodeficiency panel showed de novo heterozygous deletions of the CTLA-4 and ICOS genes. SNP microarray confirmed a de novo heterozygous 606 kb chromosome 2q33.2 deletion (204 244 976–204 851 331; GRCh37/hg19), encompassing the entire coding regions for the CTLA-4 and ICOS genes. The parents tested negative for CTLA-4 deletion. Her clinical picture was consistent with CHAI, and she was started on Abatacept 200 mg (10 mg/kg) every 4 weeks. After 9 months of therapy, she showed stable weight gain and improvement in diarrhea, appetite, nausea, vomiting, and abdominal pain. Her TPN and tube feeds were weaned. Her ESR, CRP, sIL2R, and fecal calprotectin normalized (Fig. 2) and her gross endoscopic and histopathology findings improved. She had not had severe or recurrent infections while on abatacept for almost 2 years.

The parents of the subject were aware of the intent to publish the case report and have provided verbal consent.

## DISCUSSION

Heterozygous deletions of gene clusters including CTLA-4 and ICOS have been infrequently reported,^6,7^ though deletions of this particular size have not been seen in databases of curated pathogenic copy number variants or unaffected individuals. None of these patients presented with GI manifestations only. ICOS is an autosomal recessive disease, and ICOS haploinsufficiency does not reduce the expression of ICOS on various immune cells.^6^ A heterozygous ICOS deletion is not sufficient to cause disease, whereas CTLA-4 haploinsufficiency has been associated with multiple autoimmune manifestations. Therefore, our patient’s clinical picture was consistent with CHAI.

Patients with CHAI can have autoimmune and immune dysregulation, with clinical manifestations including hypogammaglobulinemia (84%), autoimmune cytopenia (62%), severe GI symptoms (59%), respiratory disease (68%), lymphoproliferation (73%), dermatologic disease (56%), and endocrinopathies (33%). Enteropathy is the presenting symptom in about 17% of patients.^7^ Interestingly, our patient only had isolated gastrointestinal symptoms and did not have other manifestations commonly present with disease. She had IgA deficiency; however, she did not have recurrent infections or concurrent decrease in IgG, IgM, or B-cell counts commonly associated with CHAI.^7^ Her early disease onset and refractory course, rather that symptomatology, prompted further genetic testing and diagnosis.

Suspicion for monogenic IBD is high for cases with young age, poor response to conventional treatment, positive family history, immunodeficiency, autoimmunity, perianal disease, or hemophagocytic lymphohistiocytosis.^2^ Young age, alone, is a strong predictor of risk for monogenic disease, and untreated patients can have a severe and refractory course with high mortality. Therefore, this case advocates for the routine clinical practice of early genetic testing in VEO-IBD, regardless of clinical phenotype. Treatment varies according to the defect, and patients often do not respond to conventional IBD management. Likewise, our patient continued to have severe clinical symptoms on sulfasalazine but showed successful response to abatacept.

Abatacept, a CTLA-4 agonist, was used as targeted treatment for the CTLA-4 defect. Abatacept is a fusion protein composed of a fragment of the Fc domain of human IgG1 and an extracellular domain of the human CTLA-4 receptor. Similar to the CTLA-4 antigen, it competes with CD28 for binding of CD80 and CD86, therefore inhibiting costimulatory signaling and T-cell activation.^8^ It had been previously used in treatment of rheumatoid arthritis and juvenile idiopathic arthritis.^8^ In small studies and case reports, the CTLA-4 fusion proteins abatacept and belatacept have been shown to successfully control disease in patients with CTLA-4 haploinsufficiency and autoimmune enteropathy.^7,9^ In some cases, improvement in enteropathy and weight gain was seen within 3 months.^7^ sILR2, a biomarker for T-cell–mediated inflammation, had been shown to decrease with abatacept treatment, though it had been measured sporadically in prior studies.^7^ Our patient had decreasing sILR2 levels with clinical improvement. sILR2 measurement should be considered in future studies to assess its correlation with disease activity.

## References

[1] Levine A Griffiths A Markowitz J. Pediatric modification of the Montreal classification for inflammatory bowel disease: the Paris classification. Inflamm Bowel Dis. 2011; 17:3–3. 10.1002/ibd.21493 21560194

[2] Uhlig HH Schwerd T Koletzko S.; COLORS in IBD Study Group and NEOPICS. The diagnostic approach to monogenic very early onset inflammatory bowel disease. Gastroenterology. 2014; 147:3–3. 10.1053/j.gastro.2014.07.023 PMC5376484 25058236

[3] Uhlig HH Charbit-Henrion F Kotlarz D; Paediatric IBD Porto Group of ESPGHAN. Clinical genomics for the diagnosis of monogenic forms of inflammatory bowel disease: a position paper from the paediatric IBD porto group of European Society of Paediatric Gastroenterology, Hepatology and Nutrition. J Pediatr Gastroenterol Nutr. 2021; 72:3–3. 33346580 10.1097/MPG.0000000000003017 PMC8221730

[4] Zeissig S Petersen BS Tomczak M. Early-onset Crohn’s disease and autoimmunity associated with a variant in CTLA-4. Gut. 2015; 64:3–3. 10.1136/gutjnl-2014-308541 PMC4512923 25367873

[5] Kuehn HS Ouyang W Lo B. Immune dysregulation in human subjects with heterozygous germline mutations in CTLA4. Science. 2014; 345:3–3. 10.1126/science.1255904 PMC4371526 25213377

[6] Le Coz C Nolan BE Trofa M. Cytotoxic T-lymphocyte-associated protein 4 haploinsufficiency-associated inflammation can occur independently of T-cell hyperproliferation. Front Immunol. 2018; 9:3. 30087679 10.3389/fimmu.2018.01715 PMC6066513

[7] Schwab C Gabrysch A Olbrich P. Phenotype, penetrance, and treatment of 133 cytotoxic T-lymphocyte antigen 4-insufficient subjects. J Allergy Clin Immunol. 2018; 142:3–3. 10.1016/j.jaci.2018.02.055 PMC6215742 29729943

[8] Ruperto N Lovell DJ Quartier P; Paediatric Rheumatology INternational Trials Organization; Pediatric Rheumatology Collaborative Study Group. Abatacept in children with juvenile idiopathic arthritis: a randomised, double-blind, placebo-controlled withdrawal trial. Lancet. 2008; 372:3–3. 18632147 10.1016/S0140-6736(08)60998-8

[9] Lee S Moon JS Lee CR. Abatacept alleviates severe autoimmune symptoms in a patient carrying a de novo variant in CTLA-4. J Allergy Clin Immunol. 2016; 137:3–3. 26478010 10.1016/j.jaci.2015.08.036

